# Parents’ Awareness of Malocclusion and Orthodontic Consultation for Their Children: A Cross-Sectional Study

**DOI:** 10.3390/children9121974

**Published:** 2022-12-16

**Authors:** Doaa H. Alsaggaf, Manal Z. Alqarni, Shroouq A. Barayan, Abdullah A. Assaggaf, Reem A. Alansari

**Affiliations:** 1 Department of Orthodontics, Faculty of Dentistry, King Abdulaziz University, Jeddah 21589, Saudi Arabia; 2 Dental Intern, Faculty of Dentistry, King Abdulaziz University, Jeddah 21589, Saudi Arabia; 3 Dental Intern, Faculty of Dentistry, Ibn Sina National College for Medical Studies, Jeddah 22413, Saudi Arabia

**Keywords:** awareness, early diagnosis, orthodontics, parents, preventive orthodontics

## Abstract

Parents’ awareness of malocclusion and the appropriate timing to seek orthodontic consultation for their children has a substantial impact on children’s orthodontic treatment. This cross-sectional study aims to investigate parents’ awareness of malocclusion and orthodontic consultation of their children and the influence of parents’ demographics on their awareness level. An electronic survey collected information about participants’ demographics and awareness about malocclusion and orthodontic consultation of children. A total of 473 parents living in Jeddah, Saudi Arabia, participated in the study. The average awareness level among parents was moderate (3.09 ± 1.4). Male gender and unemployment were associated with low orthodontic awareness levels relative to moderate awareness (OR = 3.3, 95% CI = 1.8–5.8 and OR = 2.1, 95% CI = 1.1–3.8, respectively). Former or current orthodontic treatment was associated with high awareness levels (OR = 3.9, 95% CI = 2.4–6.5). There is still a need to raise the awareness of Saudi parents about malocclusion and orthodontic consultations for their children. Orthodontic awareness-raising campaigns should be designed to include fathers and parents with low socioeconomic status. The findings of this study also suggest that orthodontists can enhance their patient’s awareness of malocclusion and orthodontic consultation for their children.

## 1. Introduction

Orthodontics and dentofacial orthopedics is “the field of dentistry that focuses on the prevention, interception, and treatment of malocclusion, together with the skeletal and the neuromuscular abnormalities in developing or mature dentofacial structures” [1]. Orthodontic treatment, whether administered based on a professional evaluation or a patient’s self-perception, has a positive impact on children’s self-esteem and oral health-related quality of life [2,3,4]. Untreated malocclusion affects the social and emotional well-being of children [5]. It is also associated with poor school performance among adolescents [6]. Moreover, malocclusion was related to low self-esteem among adolescents, with adolescents experiencing several traits of malocclusion having considerably lower self-esteem compared to those with a single trait [7]. Some malocclusion features worsen with growth; hence, the age at which a child presents for an orthodontic consultation can be a significant factor in determining the severity of the malocclusion. This, in turn, influences the complexity of the selected treatment approach and the treatment duration of many orthodontic problems [8,9,10].

The American Association of orthodontics advises that children receive their first orthodontic consultation no later than age 7 [11]. In Saudi Arabia, results from a recently published study showed that many factors affect the decision of parents to obtain orthodontic treatment for their children such as the desire to improve facial esthetics, the perceived availability of orthodontic providers, and the cost of treatment [12]. Parents’ awareness of malocclusion and the appropriate timing for a child to seek an orthodontic consultation also has an influential role in children’s orthodontic treatment [13]. Awareness can be defined as the capability to perceive and to be conscious [14]. Several parents’ characteristics could be related to their awareness of orthodontic treatment for their children. Parents’ age, educational status, and monthly household income were significantly associated with their awareness about early orthodontic treatment for their children [15,16].

Although some previous studies have evaluated parents’ awareness of their children’s malocclusion and orthodontic treatment in Saudi Arabia, findings from previous studies mainly described the level of awareness about malocclusion and orthodontic treatment among parents [14,17,18]. It would be valuable to identify which groups of parents exhibit low awareness levels toward their children’s orthodontic treatment, particularly when allocating resources and designing orthodontic awareness-raising campaigns. Therefore, this study aims to assess the awareness of Saudi parents living in Jeddah about malocclusion and orthodontic consultation of their children, as well as the influence of demographic predictors on parents’ awareness levels. The null hypothesis for this study is that there are no significant differences in parental awareness levels of their children’s malocclusion and orthodontic consultations among Saudi parents with different demographic characteristics.

## 2. Materials and Methods

The protocol of this survey was approved by the Ethical Research Committee in the Faculty of Dentistry at King Abdulaziz University, Jeddah, Saudi Arabia (protocol code 299-10-21). An electronic survey was designed to assess parents’ awareness of malocclusion and orthodontic consultation for their children. The face validity of the questionnaire was tested by 20 parents selected randomly to evaluate the clarity and language of the questions. Content validity was also evaluated by a panel of a statistician, two dentists, and an orthodontist. The reliability of the questionnaire was ensured by sending it to 10 parents, who were asked to fill them out and return them. The same individuals completed the questionnaire after three weeks, and their responses were compared with their previous ones, which indicated excellent reliability of the questions (Cohen’s kappa statistic for the awareness survey questions ranged from 0.84 to 0.92). Detailed Cohen’s kappa statistic results for each item of the awareness questionnaire are presented in Table S1. A convenience sample of Saudi parents living in Jeddah, Saudi Arabia, was collected by distributing the survey electronically. The survey invitation stated the aims of the study explicitly and notified participants that their participation was anonymous. Participants were informed that completing the survey implied their consent and agreement to participate in the study.

### 2.1. The Questionnaire

The questionnaire included two segments. In the first segment, parents were asked about the following background questions: their age in years, gender, marital status (single, married, divorced, and widowed), current employment status (unemployed, employed, and retired), educational level (less than high school diploma, high school diploma, bachelor’s degree, and postgraduate degree). Due to few responses in some categories, the educational level variable was further grouped into a high school diploma or lower education, and a bachelor’s degree or higher education. We also collected information about the number of children, history of dental education, and history of orthodontic treatment. In the second segment, parents’ awareness of malocclusion and the appropriate timing to receive an orthodontic consultation was evaluated using the following questions:

1. Do you think a beautiful smile is important for the healthy development of a child’s personality? Answers were “Yes” or “No”.
2. What are the causes of malocclusion in children? Responses were: “Heredity”, “Bad oral habits like mouth breathing or thumb sucking”, “Both” and “I don’t know”.
3. If a primary tooth was lost prematurely due to decay. What should be done? Responses were: “Nothing. The permanent tooth will replace it”, “check if a space maintainer is needed” and “I don’t know”.
4. Who will you first go to consult regarding orthodontic treatment for your child? Responses were: “a general physician/pediatrician”, “a general dentist”, “an orthodontist” and “I don’t know”.
5. At what age should your child go to his/her first orthodontic consultation? Responses were: “7 years”, “10 years”, “13 years”, “18 years” and “I don’t know”.
6. Do you think the age of a person when starting orthodontic treatment can affect the treatment outcome? Responses were: “Yes”, “No” or “I don’t know”.
7. Do you think children can have orthodontic treatment during their growth period? Responses were: “Yes”, “No” or “I don’t know”.

A summation awareness-level variable was then constructed by dichotomizing parents’ responses to the last 6 questions into “Correct = 1” or “False = 0 with scores ranging from 0 to 6. The higher the score, the better the parent’s awareness level. Parents’ awareness levels were also categorized according to their scores into low (0–2), moderate (3–4), and high (5–6).

The sample size was calculated using Cochran’s equation based on the study by Alnaafa et al. [18]. The minimal number of individuals necessary was projected to be 369, at a margin of error = 5% and a 95% confidence interval. Data collection was conducted between December 2021 and June 2022. A total of 550 subjects were invited to participate in the study.

### 2.2. Statistical Analysis

Statistical analyses were done using SPSS version 28.0 (IBM Corp, Armonk, NY, USA). Frequency distributions were computed for parents’ demographics and responses to survey questions. The mean and frequency distribution of parents’ awareness level scores were calculated. Pearson’s chi-square tests were used to assess the association between parents’ awareness levels and their demographics. Multivariate Multinomial regression was used to predict adjusted odds ratios of parents’ awareness levels while adjusting for their background variables. A significance level of 0.05 was used in all analyses.

## 3. Results

**Figure 1:**
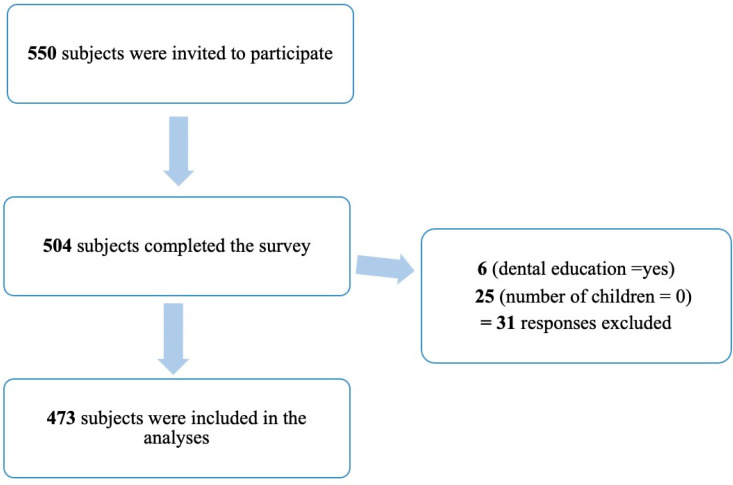
Data collection flow chart. Numbers represent the number of subjects.

Of the 550 subjects invited to participate in the study, 504 completed the survey. Responses from subjects who reported that they do not have children and parents with dental education were excluded. Thus, 473 participants were included in the analysis (Figure 1).

The average age of the participants was 37.5 ± 7.9 years. Table 1 summarizes the demographics of the survey participants. Females accounted for 57.5% of the participants. Most participants were married (92.8%), 4.7% were divorced, and 2.5% were widowed. About 33.3% of the participants had a high school diploma, and 56.9% completed their bachelor’s degree. Only 6.6% of the participants had less than a high school education, and a few had a postgraduate degree (3.6%). Nearly two-thirds of the participants were employed, while 34.9% were unemployed. About 26.6% of the participating parents had orthodontic treatment or were currently undergoing orthodontic treatment.

Most parents (97.7%) agreed that a beautiful smile is vital for a child’s good personality development (Table 2). Parents’ responses to questions about awareness of malocclusion and orthodontic consultation are shown in Table 2. Slightly more than half of the participating parents (51.6%) recognized that malocclusion in children is caused by both heredity and unhealthy oral habits. Only 38.3% of the parents were aware of the importance of space maintainers in case a primary tooth was lost prematurely, while 51.0% of them thought no action should be done in this situation. When asked about orthodontic consultations, most parents (70.2%) indicated they would visit a specialist orthodontist for an orthodontic consult for their children. However, many parents we unaware of the recommended age for a child’s first orthodontic consult, with only 28.5% choosing age 7 for their child’s first orthodontic consultation. Approximately 68.3% of the participating parents agreed that a person’s age when beginning orthodontic treatment could affect the treatment outcome. On the other hand, 52.4% of parents thought that children cannot receive orthodontic treatment while they are still growing.

**Figure 2:**
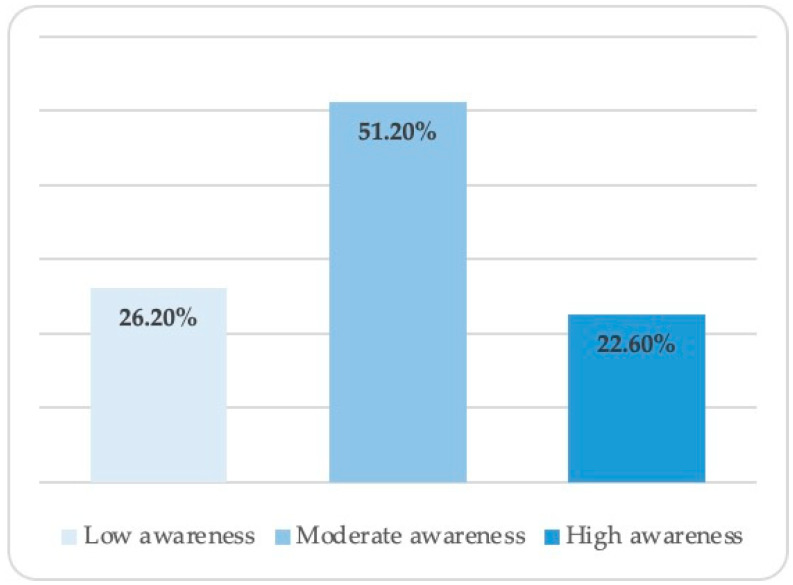
Orthodontic awareness levels among participating parents (N = 473).

By recording correct responses to the previous questions, an awareness-level variable was calculated for each parent. The mean awareness level for parents in this study was 3.09 ± 1.4. About 26.2% of parents answered 0–2 questions correctly and exhibited a low awareness level, 51.2% answered 3–4 questions correctly and showed a moderate awareness level, and 22.6% answered 5–6 questions correctly and had a high awareness level (Figure 2).

Demographic differences in parents’ awareness levels are demonstrated in Table 3. Females were significantly more likely to have better awareness levels relative to males, with 25.4% of females having high awareness levels compared to 18.9% of males. Females were also less likely to have low awareness compared to males (*p* value < 0.001). Parents with a bachelor’s degree or higher education were also more likely to have moderate and high awareness levels and less likely to have low awareness levels when compared to those with a high school diploma or a lower educational level (*p* value < 0.001). Having previous or current orthodontic treatment was significantly related to a higher awareness level among participating parents, with 41.3% of parents with previous or current orthodontic treatment experience having high awareness compared to 15.9% of those with no orthodontic treatment experience (*p* value < 0.001). Although employed parents had better awareness levels than unemployed parents, current employment status was not significantly related to parents’ awareness level (*p* value = 0.34). There were also no statistically significant differences in awareness levels among parents from different age groups, parents with different martial statuses, or a different number of children.

To further investigate the influence of demographic variables on parents’ awareness levels, multivariate multinomial regression was used. Table 4 shows demographic predictors of having low awareness levels, and high awareness levels relative to moderate awareness levels among parents. Age group, gender, employment, and previous or current orthodontic treatment remained in the final model after controlling for confounding. Compared to females, males had 3.3 times the odds of having low awareness relative to moderate awareness (OR = 3.3, 95% CI = 1.8–5.8). Males had lower yet not statistically significant odds of having high awareness than moderate awareness, compared to females (OR = 0.9, 95% CI = 0.6–1.6). The age group of parents was not significantly associated with their awareness level. Unemployed parents had 2.1 times the odds of having low awareness levels compared to employed parents (OR = 2.1, 95% CI = 1.1–3.8). However, parents’ employment status was not significantly associated with high awareness relative to moderate awareness (OR = 0.6, 95% CI = 0.4–1.2). Having previous or current orthodontic treatment was not significantly associated with low awareness relative to moderate awareness (OR = 1.2, 95% CI = 0.7–2.1). Nonetheless, parents who reported previous or current orthodontic treatment had nearly 4 times the odds of having high awareness relative to moderate awareness (OR = 3.9, 95% CI = 2.4–6.5).

## 4. Discussion

Malocclusion has adverse effects not merely on children’s facial appearance, but also on their dental health and social well-being [5,19,20]. In 2018, a study among adolescents in Jeddah, Saudi Arabia, indicated that nearly one-quarter of the examined adolescents had an extreme need for orthodontic treatment [21]. Parental awareness about malocclusion and the appropriate timing to seek an orthodontic consultation can substantially impact their child’s orthodontic treatment. This study was conducted to assess the awareness of Saudi parents living in Jeddah, Saudi Arabia, about malocclusion and orthodontic consultation for their children, and the influence of parents’ demographics on their awareness level. The findings of this study rejected the null hypothesis by showing that parents with different demographic characteristics differ significantly in their awareness about malocclusion and orthodontic consultations for their children. Our findings indicate that, on average, parents living in Jeddah have a moderate awareness level about malocclusion and orthodontic consultation for their children. Male gender and unemployment were significantly associated with low awareness relative to moderate awareness, while previous or current orthodontic treatment was associated with high awareness levels among participating parents.

In this study, 57.5% of the participating parents were females and 42.5% were males. Tabbaa et al. [17] and Alnaafa et al. [18] had a similar gender distribution among participants; however, most participants in the studies by Aldweesh et al. [16] and Basri et al. [15] were females. About 56.9% of participants in this study had a bachelor’s degree, 33% had a high school degree, and only 3.6% had a postgraduate degree. Similar to this study, more than half the participants in the studies by Tabbaa et al. [17], Alnaafa et al. [18], and Basri et al. [15] attained their bachelor’s degree. Nonetheless, studies by Tabbaa et al. [17] and Basri et al. [15] had more participants with a postgraduate education.

Most parents in the current study (97.7%) agreed that a beautiful smile is important for a child’s personality development. Tabbaa et al. reported similar findings among parents in Riyadh [17]. However, in a recent study by Aldweesh et al., a lower percentage of parents (79.35%) recognized that children’s smile esthetics impact their personality [16].

Malocclusion in children is caused by hereditary and unhealthy oral habits such as mouth breathing and digit sucking. Only 51.6% of parents in our study agreed that malocclusion is caused by hereditary and unhealthy oral behaviors, a percentage lower than that reported by Tabbaa et al. [16]. In this study, only 38.3% of parents were aware of the possible need for space maintainers to preserve the space of a prematurely lost primary tooth. Linjawi et al. also indicated low awareness about space maintainers among Saudi Adults [22].

Children are recommended to visit an orthodontist for a consultation by age 7. About 70.2% of parents in the current study reported that they would consult an orthodontist regarding orthodontic treatment for their children, and only 28.5% were aware that it is advisable for a child to get an orthodontic consultation by age 7. These findings agree with findings from Alnafaa et al. who indicated that 72.6% of parents would consult an orthodontist for their child’s need for orthodontic intervention. Their results also showed that 22.5% of parents thought the best time for an orthodontic consultation was at age 7 [18]. Basri et al. also reported that 65% of parents would take their children to the orthodontist for orthodontic treatment; however, in their study, a bigger percentage of parents (48.2%) agreed that a child’s first orthodontic screening should start at age 7 [15].

Almost two-thirds of the parents in the current study agreed that the age of a person when starting orthodontic treatment can affect the treatment outcome, and only 35.3% agreed that children can have orthodontic treatment during their growth period. Unlike our findings, most parents in Basri et al. study agreed that age is an important factor in orthodontic treatment and 74.3% of parents in their study recognized that children could have orthodontic treatment during their growth period [15]. The differences in parents’ responses between this study and Basri et al. study can be attributed to many factors, including the differences in parents’ characteristics and the differences in the way the questions were framed in the questionnaire of the two studies.

Generally, the results of this study revealed that the mean awareness level about children’s malocclusion and orthodontic consultations among parents living in Jeddah is moderate. This is in line with findings from Alnaafa et al. in Hail, Saudi Arabia [18], and Almarhoumi et al. in Al-Madinah, Saudi Arabia [23]. Despite the average moderate awareness level among parents, more than one-quarter of the participating parents had low orthodontic awareness. Al-Khalifa et al. also showed that many parents in Dammam, Saudi Arabia, had poor knowledge about preventive orthodontics [24].

When the influence of parents’ demographics on their awareness level was assessed while controlling for confounding, gender and employment status were significant predictors of low awareness levels relative to moderate awareness. Findings from Basri et al. and Almarhoumi et al. were supportive of these findings. Their studies highlighted that females and parents with a high socioeconomic status had better awareness levels [15,23]. The sole significant predictor for high awareness relative to moderate awareness among parents in this study was the history of previous orthodontic treatment or current orthodontic treatment. The odds of having high awareness levels were almost 4 times higher among parents who have had orthodontic treatment or were currently undergoing orthodontic treatment compared to those with no previous orthodontic treatment experience. In accordance with our findings, Pratelli et al. as well as Moshkelgosha et al., indicated that parents with previous orthodontic treatment experience were more likely to be solicitous of their children’s dentofacial appearance and orthodontic treatment needs [25,26]. Moreover, former orthodontic experience was associated with a positive attitude toward orthodontics [27].

The current study augments the knowledge about parents’ awareness of malocclusion and orthodontic consultations for their children. It also helps to recognize parents’ characteristics that tend to be related to their awareness levels. Parents with any dental education were excluded from the study to allow unbiased responses. Nonetheless, the findings of this study are limited and may not be generalizable to the population of Saudi Arabia as they are based on a sample from a single city.

Nowadays, as most individuals across the globe have smartphones, social media platforms could be used as means to enhance parents’ awareness of malocclusion and orthodontic consultations. Many orthodontists are using social media platforms to promote their career development and to help their patients [28]. Additionally, social media has been recently found to improve the knowledge about orthodontic treatment and oral hygiene measures among orthodontic patients [29,30]. Further research is needed to investigate the association between parental orthodontic awareness levels and their behavior and attendance to orthodontic consultations, as well as the role of social media in enriching parents’ awareness about malocclusion and orthodontic consultations of children.

## 5. Conclusions

Parents living in Jeddah exhibited a moderate awareness of malocclusion and orthodontic consultations for their children. Male gender and unemployment were associated with having low orthodontic awareness levels, while former or current orthodontic treatment was associated with high awareness levels. The findings of this study highlight that there is still a need to raise the awareness of Saudi parents about malocclusion and orthodontic consultations for their children. Orthodontic awareness-raising campaigns should be designed to include fathers and parents with low socioeconomic status. The findings also suggest that orthodontists can enhance their patient’s awareness of orthodontic treatment of their children.

## Figures and Tables

**Table 1:** Demographics of survey participants (N = 473).

Variable	n (%)
Gender	
Male	201(42.5)
Female	272 (57.5)
Age group	
18–29 years	76 (16.1)
30–39 years	204 (43.1)
40 and above	193 (40.8)
Education	
<High school diploma	31 (6.6)
High school diploma	156 (33.0)
Bachelor’s degree	269 (56.9)
Postgraduate degree	17 (3.6)
Marital Status	
Married	439 (92.8)
Divorced	22 (4.7)
Widowed	12 (2.5)
Employment	
Unemployed	165 (34.9)
Employed	308 (65.1)
Number of children	
1	85 (18.0)
2	124 (26.2)
3	95 (20.1)
≥4	169 (35.7)
Previous or current orthodontic treatment	
Yes	126 (26.6)
No	347 (73.4)

**Table 2:** Parents’ awareness of malocclusion and orthodontic consultation for their children.

Variable	n (%)
A beautiful smile is important for healthy development of the child’s personality.	
Yes	462 (97.7)
No	11 (2.3)
The causes of malocclusion in children are:	
Heredity	47 (9.9)
Bad oral habits like mouth breathing or thumb sucking	79 (16.7)
Both *	244 (51.6)
I don’t know	103 (21.8)
If a primary tooth was lost prematurely due to decay. What should be done?	
Nothing. The permanent tooth will replace it	241 (51.0)
Check if a space maintainer is needed *	181 (38.3)
I don’t know	51 (10.8)
Who will you first go to consult regarding orthodontic treatment for your child?	
General physician/pediatrician	34 (7.2)
General dentist	81 (17.1)
Orthodontist *	332 (70.2)
I don’t know	26 (5.5)
At what age should your child go to his/her first orthodontic consult?	
7 years *	135 (28.5)
10 years	56 (11.8)
13 years	113 (23.9)
18 years	77 (16.3)
I don’t know	92 (19.5)
Do you think the age of a person when starting orthodontic treatment can affect the treatment outcome?	
No	100 (21.1)
Yes *	323 (68.3)
I don’t know	50 (10.6)
Do you think children can have orthodontic treatment during their growth period?	
No	248 (52.4)
Yes *	167 (35.3)
I don’t know	58 (12.3)

* Correct answer

**Table 3:** Demographic differences in parents’ awareness of malocclusion and orthodontic consultation for their children.

Variable	Awareness Level			*p* Value
	Low	Moderate	High	
Gender				
Male	35.30%	45.80%	18.90%	<0.001
Female	19.50%	55.10%	25.40%	
Age group				
18–29 years	25.00%	57.90%	17.10%	0.32
30–39 years	29.90%	48.00%	22.10%	
40 and above	26.20%	51.80%	25.40%	
Education				
≤High school diploma	35.30%	49.70%	15.00%	<0.001
≥Bachelor’s degree	20.30%	52.10%	27.60%	
Marital Status				
Married	26.20%	50.60%	23.20%	0.1
Divorced	34.80%	43.50%	21.70%	
Widowed	9.10%	90.90%	0.00%	
Employment				
Unemployed	27.30%	53.90%	18.80%	0.34
Employed	25.60%	49.70%	24.70%	
Number of children				
1	35.30%	52.90%	11.80%	0.07
2	26.60%	49.20%	24.20%	
3	28.40%	49.50%	22.10%	
≥4	20.10%	52.70%	27.20%	
Previous or current orthodontic treatment				
Yes	19.80%	38.90%	41.30%	<0.001
No	28.50%	55.60%	15.90%	

**Table 4:** Adjusted Odds Ratios of demographic predictors for parents’ awareness levels.

	Adjusted Odds Ratio (95% Confidence Interval)		
Predictors	Low Awareness	Moderate Awareness	High Awareness
Age group		Reference	
18–29 years	Ref.		Ref
30–39 years	1.5 (0.8–2.8)		1.6 (0.8–3.5)
40 and above	1.2 (0.6–2.2)		1.7 (0.8–3.5)
Gender			
Male	Ref.		Ref.
female	3.3 (1.8–5.8) ***		0.9 (0.5–1.6)
Employment			Ref.
employed	Ref.		0.6 (0.4–1.2)
unemployed	2.1 (1.1–3.8) *		
Previous or current orthodontic treatment			
No	Ref.		Ref.
Yes	1.2 (0.7–2.1)		3.9 (2.4–6.5) ***

The reference group is moderate awareness level. * *p* value < 0.05; *** *p* value < 0.001.

## Data Availability

The data presented in this study are available on request from the corresponding author. The data are not publicly available due to privacy concerns.

## Supplementary Materials

The following supporting information can be downloaded at: https://www.mdpi.com/article/10.3390/children9121974/s1, Table S1: Kappa Coefficient for each item of the awareness questionnaire.
