# Supplementary material for: Parents’ Awareness of Malocclusion and Orthodontic Consultation for Their Children: A Cross-Sectional Study
Source: Children (Basel). 2022 Dec 16;9(12):1974. doi: 10.3390/children9121974 (PMC9776414; doi:10.3390/children9121974)
Supplement: Supplementary file 1 [file children-09-01974-s001.zip › children-2084242-supplementary.pdf]

Table S1. Kappa Coefficient for each item of the awareness questionnaire.

Question	Kappa Coefficient
A beautiful smile is important for healthy development of the child's personality.<br>Yes<br>No	0.87
The causes of malocclusion in children are:<br>Heredity<br>Bad oral habits like mouth breathing or thumb sucking<br>Both<br>I don’t know	0.89
If a primary tooth was lost prematurely due to decay. What should be done?<br>Nothing. The permanent tooth will replace it<br>Check if a space maintainer is needed<br>I don’t know	0.92
Who will you first go to consult regarding orthodontic treatment for your child?<br>General physician/pediatrician<br>General dentist<br>Orthodontist<br>I don’t know	0.86
At what age should your child go to his/her first orthodontic consult?<br>7 years<br>10 years<br>13 years<br>18 years<br>I don’t know	0.84
Do you think the age of a person when starting orthodontic treatment can affect the treatment outcome?<br>No<br>Yes<br>I don’t know	0.80
Do you think children can have orthodontic treatment during their growth period?<br>No<br>Yes<br>I don’t know	0.88
